# Bronchial Fibroblasts from Asthmatic Patients Display Impaired Responsiveness to Direct Current Electric Fields (dcEFs)

**DOI:** 10.3390/biomedicines11082138

**Published:** 2023-07-29

**Authors:** Anastasiia Pavlenko, Sławomir Lasota, Dawid Wnuk, Milena Paw, Jarosław Czyż, Marta Michalik, Zbigniew Madeja

**Affiliations:** Department of Cell Biology, Faculty of Biochemistry, Biophysics and Biotechnology, Jagiellonian University, Gronostajowa 7, 30-387 Kraków, Poland; nastia.netychuk@gmail.com (A.P.); dawid.wnuk@uj.edu.pl (D.W.); milena.paw@uj.edu.pl (M.P.); jarek.czyz@uj.edu.pl (J.C.); marta.michalik@uj.edu.pl (M.M.)

**Keywords:** electrotaxis, galvanotaxis, human bronchial fibroblasts, asthma

## Abstract

Accumulating evidence suggests that an important role is played by electric signals in modifying cell behaviour during developmental, regenerative and pathological processes. However, their role in asthma has not yet been addressed. Bronchial fibroblasts have recently been identified having important roles in asthma development. Therefore, we adapted an experimental approach based on the lineages of human bronchial fibroblasts (HBF) derived from non-asthmatic (NA) donors and asthmatic (AS) patients to elucidate whether their reactivity to direct current electric fields (dcEF) could participate in the asthmatic process. The efficient responsiveness of NA HBF to an electric field in the range of 2–4 V/cm was illustrated based on the perpendicular orientation of long axes of the cells to the field lines and their directional movement towards the anode. These responses were related to the activity of TGF-β signalling, as the electrotaxis and re-orientation of NA HBF polarity was impaired by the inhibitors of canonical and non-canonical TGF-β-dependent pathways. A similar tendency towards perpendicular cell-dcEF orientation was observed for AS HBF. However, their motility remained insensitive to the electric field applied at 2–4 V/cm. Collectively, these observations demonstrate the sensitivity of NA HBF to dcEF, as well as the inter-relations between this parameter and the canonical and non-canonical TGF-β pathways, and the differences between the electrotactic responses of NA and AS HBF point to the possible role of their dcEFs in desensitisation in the asthmatic process. This process may impair the physiologic behaviour of AS HBF functions, including cell motility, ECM deposition, and contractility, thus promoting bronchial wall remodelling, which is a characteristic of bronchial asthma.

## 1. Introduction

The presence of endogenous direct current electric fields (dcEFs) within extracellular spaces of tissues was detected for the first time more than 170 years ago [1]. However, only in recent decades have numerous studies demonstrated the crucial role played by electric signals in the regulation of cell behaviour during developmental, regenerative, and pathological processes [2,3,4]. Most commonly, they participate in these processes by guiding cell migration in a process known as electrotaxis or galvanotaxis, i.e., a directional movement towards the cathode or anode. In vitro application of physiological strength dcEF was found to induce electrotaxis in a variety of cultured cells [5]. Most of the cell types migrate towards the cathode, including human retinal pigment epithelial cells [6]; human keratinocytes [7,8,9,10]; bovine, rabbit, and human corneal epithelial cells [11,12,13]; bovine aortic vascular endothelial cells [14]; neonatal human dermal fibroblasts (nHDFs) [15]; amphibian and avian neural crest cells [16,17,18]; fish epidermal cells [19,20,21]; rat carcinosarcoma WC256 cells [22]; and metastatic rat prostate cancer cells [4]. On the other hand, rabbit osteoclasts [23], murine resident peritoneal macrophages [24], human granulocytes [25], human-induced pluripotent stem (hiPS) cells [26] and human umbilical vein endothelial cells (HUVECs) were found to migrate towards the anode [27]. It is assumed that the quality and quantity of electrotaxis is determined based on cellular phenotype and the tissue-specific context.

dcEFs in epithelial tissues are generated by the directional transport of ions across epithelial layers as a result of the polarised distribution of membrane ion channels and transporters. Consequently, polarised ion transport leads to the creation of a transepithelial potential (TEP) [2,3]. After rupture of the epithelium, TEP collapses at the wound centre but remains stable distally. The resulting voltage gradient establishes the dcEF with a vector parallel to the epithelial surface and the wound centre as the cathode [28]. Such dcEFs with a magnitude of 0.4–2 V/cm were detected at mammalian skin wounds [29]. They guide the electrotaxis of keratinocytes towards the wound centre (cathode). During the healing of the wound, epidermal cells cooperate with fibroblasts that support the whole process by triggering wound contraction and depositing new extracellular matrix (ECM) [30]. Previous studies demonstrated that fibroblasts respond to dcEF by active directional migration toward the cathode or anode. Consequently, dcEF promotes wound healing by the cooperative induction of re-epithelialisation and the activation of stromal fibroblasts that includes directional movement, the secretion of ECM and pro-myofibroblastic transformation [31].

Endogenous dcEF may also be important for wound healing and the restitution of the respiratory system [32]. Respiratory epithelia generate considerable TEPs [33,34] that generate lateral EFs after injury, inducing the electrotaxis of airway epithelial cells. Epithelial dynamics, differentiation, and regeneration also seem to play prominent roles in the asthmatic process [35]. Asthma is a chronic inflammatory disease that is defined as a series of chronic structural changes that lead to epithelial damage, subepithelial fibrosis, increased accumulation of extracellular matrix (ECM), smooth muscle hypertrophy, thickening of the airway wall, and increased vascularity [36]. Growing evidence is accumulating that the dysregulated epithelial repair of the airway wall is present in asthma [37]. For example, asthmatic airway epithelial cells were shown to exhibit impaired wound healing [38]. Furthermore, dysregulated differentiation of asthmatic epithelium allows repair, but not proper regeneration, following injury [39]. Consequently, it was suggested that, as observed in asthma, airway inflammation and remodelling occur as a result of increased susceptibility to injury and impaired wound healing [40]. It is conceivable that dcEF may also be involved in asthma development. On the other hand, the role of dcEF in asthma development has not yet been considered.

Damage to the epithelium during airway remodelling is accompanied by the activation of neighbouring fibroblasts in the process of fibroblast-to-myofibroblast transition (FMT). Our previous studies have shown that some inherent characteristics of fibroblasts can play a significant role in the FMT. Differences observed between AS and NA HBF concern, in particular, their susceptibility to TGF-β-induced FMT [41,42,43,44,45,46]. In addition to their different reactivity to TGF-β, asthmatic (AS) and non-asthmatic (NA) human bronchial fibroblasts (HBF) also show differences in terms of cell shape, elasticity, and protein expression profile [36,47,48]. However, these differences have not yet been considered in terms of their sensitivity to dcEFs. Therefore, to outline the role of physiologic dcEFs in the asthmatic process, we evaluated the following aspects: (i) the reactivity of NA HBF to dcEFs, (ii) its potential links to canonical/non-canonical TGF-β signalling, and (iii) its relationship with the pro-asthmatic phenotype of AS HBF.

## 2. Materials and Methods

### 2.1. Cell Culture

Human bronchial fibroblasts (HBF), from asthmatic patients (AS)—isolated as described previously [42], or donors with asthma excluded (NA) (Lonza, CC-2512, Lonza Group AG, Basel, Switzerland). Cells were cultured in a ‘complete culture medium’—DMEM HG (Dulbecco’s modified Eagle’s Medium High Glucose), with 10% FBS (Foetal Bovine Serum) and both penicillin (100 I.U./mL)/streptomycin (100 μg/mL) at 37 °C and 5% CO_2_. Cells were routinely passaged at the confluence of 80%.

### 2.2. Application of Electric Field

The direct current electric field was applied using a custom-made electrotactic chamber and the protocol described earlier [49], albeit with minor modifications. An observation chamber made of two glasses that measured 60 × 35 × 0.2 mm separated by two glasses that measured 60 × 10 × 0.2 mm, with all glasses connected by double-sided adhesive tape (tesa SE, Hamburg, Germany), was mounted in the PVC chamber and sealed with silicon paste. Cells were seeded in the incomplete observation chamber (without top glass) 24 h prior to the experiment at a density of 2.5 × 10^3^ per cm^2^ in 400 μL of complete culture medium and kept in the humidity chamber. During chamber assembly, the culture medium was replaced with an analogous medium that lacked sodium bicarbonate. The new medium contained HEPES buffer (15 mM), and the pH was set at 7. This medium was later referred to as the ‘recording medium’. Ag/AgCl electrodes (6 cm^2^ each) immersed in PBS, which were connected to the power supply, were used to apply a dcEF of 1 to 4 V/cm. Continuous monitoring of EF intensity was made possible by the use of two measuring electrodes located near both openings of the observation chamber.

### 2.3. Registration of Cell Migration and Morphology

Time-lapse imaging was performed at integrated modulation contrast (IMC) using a fully motorised Leica DMi8 microscope equipped with an MC170-HD CMOS camera and HC FL PLAN 10×/0.25 DRY objective controlled via LAS X 3.7 software (all Leica, Wetzlar, Germany). A temperature of 37 °C was maintained inside the observation chamber (described earlier) using an environmental chamber and a heating unit (PeCon GmbH, Erbach, Germany). The images were captured every 10 min for 8 h and 30 min, with EF application occurring after an initial 30-min period (with the cathode at the right-hand side).

### 2.4. Analysis of Cell Migration

Single-cell migration analysis was performed using the method previously described [50,51] via Hiro 1.0.0.4 software (written by Wojciech Czapla, Kraków, Poland). Cell migration trajectories were constructed from cell centroids that were manually determined. To obtain circular diagrams, the initial point of each trajectory was placed in the origin of the coordinate system. The following quantitative parameters were calculated: (a) the speed of cell migration (μm/min)—the total length of the cell trajectory divided by time of recording; (b) cell displacement (μm)—the length of line segment from the first to the last position of a cell; (c) average directional cosine γ—γ is the angle formed by the line that connected the initial point of trajectory to subsequent cell positions and the *X*-axis (parallel to the vector of EF).

### 2.5. Analysis of Cell Orientation

Outlines of cells manually created via the Fiji ImageJ 1.53m program (National Institutes of Health, Bethesda, MD, USA) [52] were used to assess the orientation of the long axis of cell to the *X* axis (parallel to the vector of EF, if applied). Values designated using the program were reduced to angle α (ranging from 0 to 90 for parallel to perpendicular orientation, respectively), and the orientation index was subsequently calculated using the formula (α-45)/45, resulting in a value range of −1 to 1 (for parallel and perpendicular orientation to EF, respectively). Additionally, the value 0 within cell population, on average, denotes random orientation relative to the *X*-axis.

### 2.6. Inhibition of Signalling Pathways

Particular components of TGF-β receptor signalling pathways were pharmacologically inhibited. Specifically, recording medium that contained SB431542 (for the TGF-β receptor I itself), U0126 (for MEK1/2), SB203580 (for p38), or Y-27632 (for ROCK) (all Sigma-Aldrich, St. Louis, MO, USA) at a concentration of 10 μM (as described earlier [53]) was provided to cells during electrotactic chamber assembly, with this process occurring 30 min prior to the start of time-lapse imaging and being present throughout the experiment.

### 2.7. Induction of the Fibroblast-to-Myofibroblast Transition (FMT)

Each cell population was seeded in two wells of silicone culture inserts (Ibidi GmbH, Gräfelfing, Germany) that were placed onto a single glass of the observation chamber (measuring 60 × 35 × 0.2 mm) at a density of 2.5 × 10^3^/cm^2^ in complete culture medium. After 24 h, the medium was replaced with serum-free DMEM HG medium that contained 0.1% BSA. After another 24 h, TGF-β was added (human natural, Corning Incorporated, Corning, NY, USA; (356040)) at a final concentration of 5 ng/mL. Control cells in the neighbouring compartment remained untreated. After 5 days of incubation, dcEF application and time-lapse imaging were performed using the method described earlier. Cells that presented a phenotype of myofibroblasts (i.e., well-spread cells with significantly larger areas) were selected for quantitative analysis.

### 2.8. Immunofluorescent Staining and Fluorescence Microscopy

Myofibroblasts were detected via α-SMA immunofluorescence staining. Cells were seeded at a density of 5.0 × 10^3^/cm^2^ in a 12-well plate that contained sterile cover glasses. FMT induction was conducted via the method previously described [42]. Cells were fixed with 3.7% formaldehyde in PBS for 15 min and permeabilised with 0.1% Triton X-100 for 10 min (both steps occurred at room temperature). The non-specific binding sites were blocked with 1% BSA in PBS (Sigma-Aldrich) for 45 min, followed by overnight incubation with mouse monoclonal antibody anti-α-SMA in 1% BSA (Sigma-Aldrich, A2547, 1:400). Next, the cover glasses were incubated with secondary goat anti-mouse antibody conjugated with AlexaFluor 488 (A-11001, 1:500, Invitrogen, Waltham, MA, USA) and Hoechst 33258 (Sigma-Aldrich, 1 μg/mL), which all occurred in 1% BSA for 1 h. Finally, cellular microscope slides were embedded in a fluorescence mounting medium (Dako, Glostrup, Denmark). Imaging was performed via a fully motorised fluorescence microscope Leica DMI6000B, which was equipped with a DFC360FX monochromatic CCD camera and HCX PL APO 40×/1.25 OIL immersion objective, as well as controlled via LAS X 3.4 (all Leica, Wetzlar, Germany).

### 2.9. Statistical Analysis

The statistical significance of differences in the speed of cell migration and cell displacement were assessed via Student’s *t*-test, while for directionality and orientation, these differences were assessed via a non-parametric U–Mann–Whitney test using Statistica 13 software (TIBCO Software Inc., Palo Alto, CA, USA) due to the nature of these parameters and the inherent lack of a normal distribution associated with them. Statistical significance was determined based on *p* < 0.05.

## 3. Results

### 3.1. Electrotactic Responses of NA HBF to dcEFs

**Figure 1:**
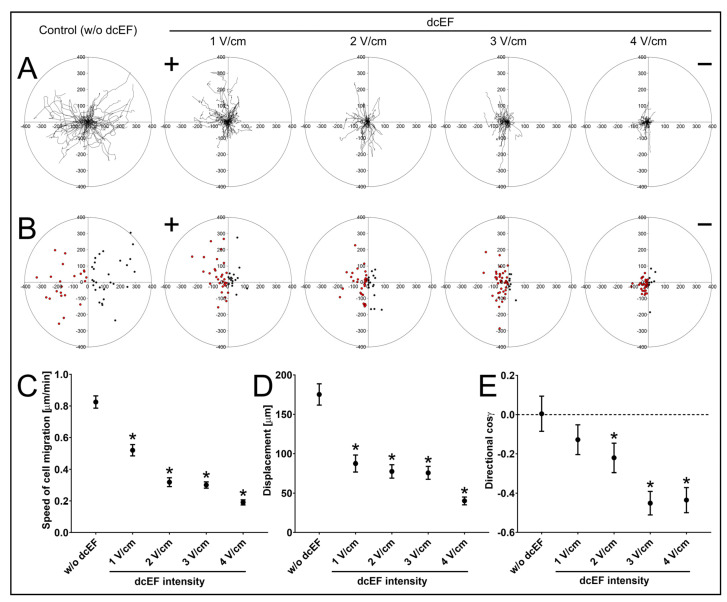
Migration of NA HBF in isotropic conditions and an electric field of physiological magnitude. (**A**) Composite trajectories of NA HBF migration both without and in the presence of a direct current electric field (dcEF) of physiological magnitude (1–4 V/cm) are shown as circular diagrams. The initial point of each trajectory was moved to the origin of the coordinate system. Each trajectory was created using 48 points of cell centroids recorded at 10-minute intervals for 8 h. The positive electrode (anode) of dcEF (if present) is located on the left side. Scale in μm; *n* = 50 cells per condition. (**B**) Final positions of NA HBF migration plotted on the coordinate system. dcEF (if present) is oriented as previously. Points located on the left (anodal) side of the 0Y axis are marked in red, while points located on the right (cathodal) side of the 0Y axis are marked in black. Scale in μm; *n* = 50 cells per condition. (**C**–**E**) The quantitative parameters of NA HBF migration both without and in the presence of dcEF. (**C**) Speed of cell migration; (**D**) cell displacement; (**E**) directionality of cell migration. Values are calculated as the mean value for the analysed population ± SEM (*n* = 50); * statistically significant values relative to those of control (*p* < 0.05).

Electrotaxis is the most prominent dcEF-induced cellular response. Therefore, to estimate the electrotactic responsiveness of NA HBF, we first analysed their motility after exposition to dcEFs applied at 1–4 V/cm (Table 1; Figure 1). In the absence of an external electric field, NA HBF showed random directionality in cell movement. At the single-cell level, this observation was illustrated by the circular diagrams that show the distribution of single-cell trajectories (Figure 1A) and displacements (Figure 1B). At the population level, the random directionality of NA HBF movement is shown by the value of cosine γ (0.004 ± 0.091; Table 1; Figure 1E). Following the application of an electric field, NA HBF movement became strongly directional towards the anode. The increase in dcEF strength significantly enhanced the directionality of cell movement, as illustrated by cosine γ values (Table 1; Figure 1E). These data demonstrate the significant reactivity of NA HBF to dcEFs and suggest their involvement in bronchial healing and remodelling. On the other hand, the induction of NA HBF electrotaxis was accompanied by a significant decrease in other motility parameters, including the speed of cell migration and displacement, that occurred in the presence of dcEF (Table 1; Figure 1C,D). This effect was probably related to a change in the orientation of the long axes of the migrating NA HBF in the electric field (see below).

### 3.2. The Effect of an Electric Field on the Orientation of the Long Axis of NA HBF

**Figure 2:**
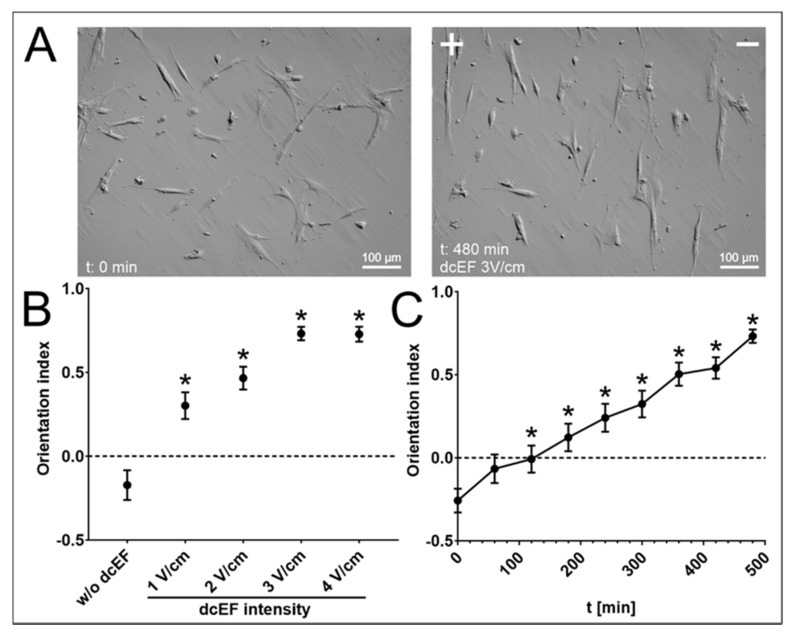
Orientation of NA HBF in dcEF compared to isotropic conditions. (**A**) Cell morphology before and after 8 h of application of 3 V/cm of dcEF. The positive electrode (anode) of dcEF (if present) is located on the left side of the field of view. (**B**) Orientations of the long axes of NA HBF in relation to the electric field vector (and EF lines). Mean orientation index for cell population (*n* = 50) after 8 h of exposure to EF of specified strength (1–4 V/cm) ± SEM. A value of 0 denotes random orientation, while a value of 1 denotes perpendicular orientation and a value of −1 denotes parallel orientation of the long axes of cells to EF lines. * statistically significant values compared to those of the control (*p* < 0.05) (**C**) Change in orientation index over time. 3 V/cm of dcEF was applied to NA HBF for 8 h, and cell orientation was assessed every hour as the mean value ± SEM (*n* = 50). * statistically significant values compared to the initial time point (*p* < 0.05).

To elucidate the processes that drove the inhibition of the NA HBF speed of movement during the electrotaxis, we analysed the effect of dcEF (applied at 1–4 V/cm) on the orientation of the long axes of NA HBF in relation to the lines of the electric field. When cultivated in control conditions, no predominant orientation of the NA HBF axes could be seen. This finding is illustrated by photomicrographs and data from morphometric analyses that gave an orientation index value (O_i_) close to 0 (O_i_ equal to 1 means that every cell is perfectly perpendicularly aligned relative to the electric field lines, while O_i_ equal to −1 denotes the cells’ parallel orientation to the electric field lines; Figure 2A(left),B). dcEF application induced the reorientation of cellular axes, meaning that their alignment was mostly perpendicular to the electric field lines (Figure 2A(right)). This response was dcEF strength-dependent, and its greatest magnitude was observed for NA HBF exposed to 3 V/cm of dcEF (Figure 2B), where the average O_i_ reached 0.731 ± 0.040. In turn, its time dependence was demonstrated using O_i_ dynamics, which increased with the duration of the experiment (Figure 2C). Collectively, these data confirm the sensitivity of NA HBF to dcEFs. They also show that electric fields may affect arrangement of fibroblasts at the periphery of the wound.

### 3.3. TGF-β Signalling Participates in NA HBF Responses to dcEFs

TGF-β is a crucial cytokine for the remodelling of bronchial walls during the asthmatic process. Moreover, it was suggested that directed cell migration in the electric field is dependent on TGF-β [13]. In this model, asymmetric signalling results from the increase in the density of TGF-β membrane receptors on one side of the cell, while the concentration of TGF-β (present in serum) around the cell remains uniform.

**Figure 3:**
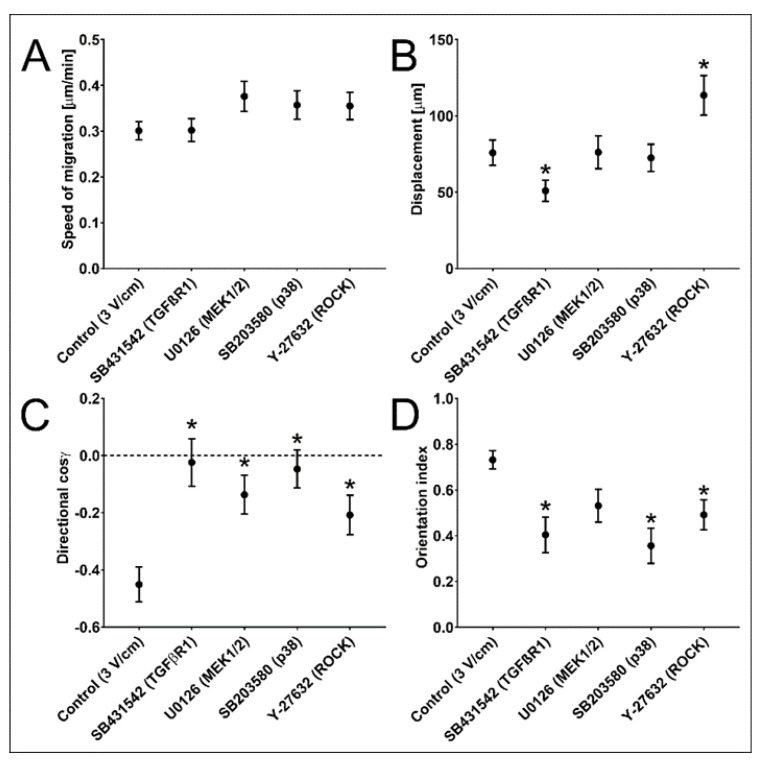
The effect of pharmacological inhibition of TGF-β signalling on the response of NA HBF to the dcEF. (**A**–**D**) The quantitative parameters of NA HBF migration and orientation in a 3 V/cm dcEF applied for 8 h both without and in the presence of particular inhibitors of TGF-β signalling pathways. The names of the inhibitors and targeted proteins are listed under the *X* axis. Each inhibitor was used at a concentration of 10 μM. (**A**) Speed of cell migration; (**B**) cell displacement; (**C**) directionality of cell migration; (**D**) orientation of the long axis in relation to EF lines; mean ± SEM (*n* = 50); * statistically significant value compared to that of the control (*p* < 0.05).

Therefore, we further elucidated whether TGF-β signalling determines the susceptibility of NA HBF to dcEFs. To address this point, we used specific inhibitors relevant to the canonical (TGF-β receptor I inhibitor: SB431542; 10 μM) and non-canonical (p38 MAPK inhibitor: SB203580; MEK inhibitor: U0126; ROCK inhibitor: Y-27632; 10 μM) TGF-β signalling pathways. All investigated inhibitors impaired the electrotactic responses of NA HBF to 3 V/cm of dcEF (Table 2, Figure 3C). This observation is illustrated by changes in the direction of cosine values calculated for NA HBF exposed to 3 V/cm of dcEF in their presence (Figure 3C). Considerable differences between their effects on other NA HBF responses are illustrated based on a moderate impairment of the NA HBF displacement in the presence of 3 V/cm of dcEF and SB431542 (inhibitor of TGF-β receptor I; ALK4, ALK5 and ALK7: to the 67% of the control value; 50.99 ± 6.97 μm vs. 75.80 ± 8.27 μm). On the other hand, ROCK inhibitor Y-27632 significantly stimulated cell migration (150% of the displacement control value; 113.53 ± 12.97 μm vs. 75.80 ± 8.27 μm) (Figure 3B). The inhibitors of canonical and non-canonical TGF-β signalling pathways exerted corresponding effects on the orientation of NA HBF in dcEF. Our data show the significance of the interplay between downstream effectors of TGF-β-dependent pathways in enabling the fine-tuning of electrotactic cell responses to dcEFs.

### 3.4. AS HBF Display Aberrant Reactivity to dcEFs

**Figure 4:**
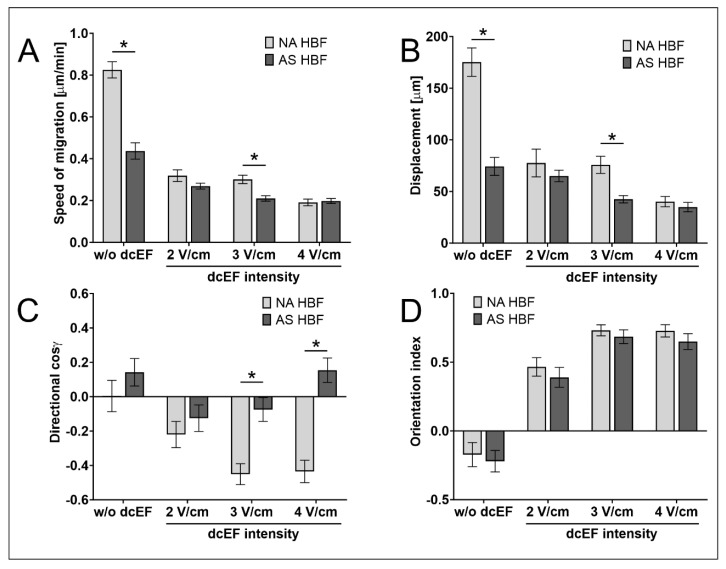
Reactivity of human bronchial fibroblasts derived from asthmatic patients (AS HBF) to an electric field. (**A**–**D**) The quantitative parameters that defined the migratory activity and orientation of AS HBF compared to NA HBF both without and in the presence of a direct current electric field (dcEF) of physiological magnitude (2–4 V/cm) which were applied for 8 h. (**A**) Speed of cell migration; (**B**) cell displacement; (**C**) directionality of cell migration; (**D**) orientation of the long axis in relation to EF lines. Mean values for the cell population (*n* = 50) ± SEM. * statistically significant differences between AS and NA (*p* < 0.05).

As our data show the sensitivity of NA HBF to external dcEF, we further concentrated on its involvement in the asthmatic process. For this purpose, we used an experimental approach based on the HBF lineages derived from asthmatic (AS) patients and traced the differences between the reactivity of AS and NA HBF to dcEFs. Whereas AS HBF showed a clear tendency to assume a perpendicular orientation of the long axis of the cell in the electric field (similarly to NA HBF, Table 3; Figure 4D), considerable differences could be seen in their motile activity and electrotactic response (Table 3; Figure 4A–C). 

In particular, no electrotactic reaction of AS HBF could be observed in the presence of dcEFs applied at 2–4 V/cm (Figure 4C). This observation extends the list of differences between these two cell populations to include different reactivity to physiological electric fields.

**Figure 5:**
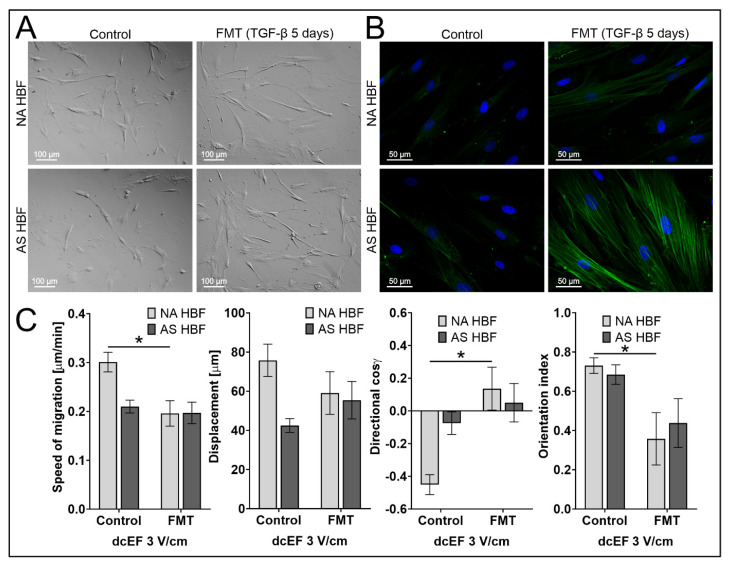
Reactivity of myofibroblasts derived from NA and AS HBF to an electric field. (**A**–**B**) AS HBF are compared to NA HBF before and after 5 days of stimulation with TGF-β (5 ng/mL) to induce a fibroblast-to-myofibroblast transition (FMT). (**A**) Images of cell morphology obtained via modulation contrast. Scale bar—100 μm. (**B**) Immunodetection of α-SMA (green). Images obtained via fluorescence microscopy. Cells are counterstained for cell nuclei (blue). Scale bar—50 μm. (**C**) The quantitative parameters that define the migratory activities and orientations of AS HBF and NA HBF that underwent FMT both without and in the presence of a 3 V/cm dcEF applied for 8 h. Mean values for the cell population ± SEM. * statistically significant differences between myofibroblasts (*n* = 20) and undifferentiated (*n* = 50) cells (*p* < 0.05).

Mechanistic studies revealed that AS HBF were much more susceptible than NA HBF to differentiation into myofibroblasts after TGF-β stimulation in serum-free media (Figure 5A,B). Notably, we also observed a significant effect of TGF-β-induced fibroblast–myofibroblast transition (FMT) on their susceptibility to dcEFs. The ability of NA HBF to respond to the electric field was markedly impaired after their differentiation into myofibroblasts (Table 4; Figure 5C). Its magnitude was reduced to the values characteristic for AS HBF. Although our data indicate that the TGF-β present in the serum is necessary for the induction of electrotactic NA HBF responses, after TGF-β induced differentiation of fibroblasts into myofibroblasts, their ability to react in response to the presence of electric fields decreases and remains at a similar level for cells derived from asthmatic patients.

## 4. Discussion

Respiratory epithelium has been found to generate the TEP that is responsible for the creation of lateral dcEF after injury [32,33,34], which affects directional migration of airway epithelial cells. Therefore, it was reasonable to assume that dcEF might also affect the bronchial fibroblasts present in the wound area, whereas electrotactic AS HBF aberrations might be responsible for initiating the asthmatic process. Surprisingly, this point has not yet been addressed. Our study is the first project to demonstrate the following points: (i) that adult human bronchial fibroblasts are sensitive to dcEFs, (ii) this sensitivity depends on TGF-β activity, and (iii) AS HBF display aberrant reactivity to dcEFs. Apparently, these findings may account for bronchial wall remodelling that occurs during the asthmatic process.

The reactivity of human bronchial fibroblasts, which responded efficiently to dcEF, is consistent with observations made in other laboratories. For example, an anodal electrotactic response to dcEFs has been reported for the foetal human lung fibroblasts MCR-5 [54], cardiac fibroblasts (CFs) [55], human skin fibroblasts [31,56], and mouse skin fibroblasts [57]. However, the reactivity of fibroblasts to dcEF is controversial. For example, it was reported that C3H/10T1/2 mouse embryo fibroblasts [58], neonatal human dermal fibroblasts (nHDFs) [15], mouse embryonic 3T3 fibroblasts [59,60,61], mouse skin fibroblasts [62], and embryonic quail somite fibroblasts [63] migrated towards the cathode, while human gingival fibroblasts [64] and human dermal fibroblasts [65] did not respond to EF. Also, the perpendicular orientations of the long axes of the cells relative to the electric field lines have previously been observed for the fibroblasts derived from other tissues [57,58,60,63]. Our study was the first project to report such a response in bronchial fibroblasts. It can be speculated that the dcEF induced migration of fibroblasts to the periphery of the wound (toward the anode) and their uniform arrangement in relation to the electric field lines may have a positive effect on wound contraction and ultimately accelerate wound healing.

Our study was also the first project to link disturbed electrotaxis of bronchial fibroblasts to the asthmatic process. Our previous papers reported on the differences observed between AS and NA HBF, such as by determining the reactivity of these fibroblasts to TGF-β, cell shapes, elasticity and contractility, and protein expression profiles [41,42,43,44,45,47]. Here, we showed that HBF populations derived from AS patients and NA donors showed the same tendency towards perpendicular alignment of the long axes of the cells relative to the field lines (Figure 4). However, unlike NA HBF, AS HBF do not show electrotactic reactions in an electric field of 2–4 V/cm. The results of this report extend the list of differences between AS and NA HBF and indicate that different reactivity to physiological electric fields may participate in aberrant bronchial remodelling and the development of asthma.

Despite performing an intensive research, the mechanism of electrotaxis was not fully elucidated. One of the proposed hypotheses used to explain this process assumed that membrane receptors for chemoattractants are moved to one side of the cell via EF and direct cell migration [2]. Such a mechanism has been demonstrated for the EGF receptor and, to lesser extent, for the FGF and TGF-β receptors [13,66]. For instance, the cathodal electrotactic response and perpendicular orientation of bovine corneal epithelial cells were TGF-β dependent [13]. However, it was not investigated whether this reaction was related to TGF-β-induced canonical or non-canonical signalling. TGF-β is one of the main mediators involved in the regulation of fibroblast differentiation and tissue remodelling in the bronchi. In our hands, the inhibitors of both canonical and non-canonical TGF-β signalling pathways efficiently weakened electrotaxis and the morphologic reorientation of NA HBF in dcEFs, indicating that activity of these pathways is necessary for NA HBF electrotaxis. It was previously demonstrated that RhoA activity controls the perpendicular orientation of mouse skin fibroblasts in response to dcEF [57]. In this study, inhibiting ROCK (the main RhoA effector) resulted in a significant decrease in both the orientation index and directionality of HBF migration. On the other hand, although p38 activity was found to be essential in generating the directional response to dcEF identified in this study, its crucial role in the electrotaxis of human fibrosarcoma and glioma cells was ruled out [67,68]. In addition to short-term effects, human bronchial fibroblasts drive the fibroblast-to-myofibroblast transition (FMT) in response to transforming growth factor-β (TGF-β), and this reaction depends both on canonical Smad-dependent signalling [41,42,43,44,45] and non-canonical MAPK signalling [53]. As NA HBF loses the ability to perform anodal electrotaxis after FMT, this finding may suggest that TGF-β-induced differentiation brings the phenotype closer to that of AS HBF, which is non-responsive to EF in terms of directional migration.

It should be mentioned that although TGF-β is only one of the cytokines involved in the control of cell migration, as discussed in the literature above, it may serve as the connecting point between electrotaxis and airway remodelling that occurs in asthma. However, to obtain a more in-depth understanding of HBF electrotaxis, other cytokines (including other growth factors) should be examined in further research. In particular, the role of the EGF receptor and its signalling pathways could be assessed due to its robust redistribution in the dcEF. The results presented in this study have another limitation: all experiments were conducted using rigid 2D substrates. It can be expected that an elastic substratum or 3D conditions based on collagen gel could affect both the migratory activity and FMT efficiency of the examined cells. However, it is worth noting that mouse skin fibroblasts presented similar electrotaxis and perpendicular orientations to dcEF lines in both 2D conditions and when embedded in a collagen matrix [57]. Therefore, we hope that the results obtained in this study could be reflected in more physiological conditions. Such a model would be particularly convenient, especially since certain cells (such as human fibrosarcoma HT1080) demonstrate sensitivity to weaker dcEF when placed in 3D conditions [69], suggesting an even more robust response than that recorded in our cellular model.

In conclusion, we have demonstrated for the first time that adult human bronchial fibroblasts respond to physiologically tuned external electric fields. dcEFs induce cell orientation that is perpendicular to the field lines and efficient electrotaxis in the direction of the anode. These processes depended both on canonical and non-canonical TGF-β pathways. Differences between the electrotactic responses of fibroblasts sourced from asthmatic (AS) patients and non-asthmatic (NA) donors may be crucial to subepithelial fibrosis during the remodelling of the bronchial wall during the asthmatic process.

## Figures and Tables

**Table 1:** Migration of NA HBF in isotropic conditions and an electric field of physiological magnitude.

NA HBF; *n* = 50	Speed of Cell Migration [μm/min]	Cell Displacement [μm]	Directionality (cos γ) [No Unit]
dcEF Intensity	Mean ± SEM	Mean ± SEM	Mean ± SEM
w/o (Control)	0.825 ± 0.039	175.248 ± 13.693	0.004 ± 0.091
1 V/cm	0.520 ± 0.036 *	87.540 ± 10.835 *	−0.128 ± 0.077
2 V/cm	0.319 ± 0.028 *	77.610 ± 13.450 *	−0.220 ± 0.076 *
3 V/cm	0.301 ± 0.020 *	75.796 ± 8.270 *	−0.451 ± 0.061 *
4 V/cm	0.191 ± 0.016 *	40.143 ± 4.967 *	−0.435 ± 0.065 *

* statistically significant values relative to those of the control (*p* < 0.05).

**Table 2:** The effect of pharmacological inhibition of TGF-β signalling on the response of NA HBF to the dcEF.

NA HBF; 3 V/cm dcEF; *n* = 50	Speed of Cell Migration [μm/min]	Cell Displacement [μm]	Directionality (cos γ) [No Unit]	Orientation Index [No Unit]
Inhibitor (Target Protein)	Mean ± SEM	Mean ± SEM	Mean ± SEM	Mean ± SEM
w/o (Control)	0.301 ± 0.020	75.796 ± 8.270	−0.451 ± 0.061	0.731 ± 0.040
SB431542 (TGFβR1)	0.302 ± 0.025	50.990 ± 6.972 *	−0.024 ± 0.083 *	0.404 ± 0.078 *
U0126 (MEK1/2)	0.376 ± 0.033	76.163 ± 10.766	−0.137 ± 0.068 *	0.531 ± 0.072
SB203580 (p38)	0.357 ± 0.031	72.527 ± 8.941	−0.047 ± 0.066 *	0.356 ± 0.077 *
Y-27632 (ROCK)	0.355 ± 0.030	113.532 ± 12.974 *	−0.208 ± 0.069 *	0.492 ± 0.065 *

* statistically significant value compared to that of the control (*p* < 0.05).

**Table 3:** Reactivity of human bronchial fibroblasts derived from asthmatic patients (AS HBF) to an electric field.

AS HBF; *n* = 50	Speed of Cell Migration [μm/min]	Cell Displacement [μm]	Directionality (cos γ) [No Unit]	Orientation Index [No Unit]
dcEF Intensity	Mean ± SEM	Mean ± SEM	Mean ± SEM	Mean ± SEM
w/o (Control)	0.437 ± 0.039 *	74.273 ± 8.689 *	0.142 ± 0.080	−0.220 ± 0.078
2 V/cm	0.269 ± 0.014	64.999 ± 5.579	−0.125 ± 0.077	0.389 ± 0.072
3 V/cm	0.210 ± 0.013 *	42.496 ± 3.504 *	−0.075 ± 0.069 *	0.685 ± 0.050
4 V/cm	0.198 ± 0.012	34.867 ± 4.502	0.154 ± 0.071 *	0.649 ± 0.057

* statistically significant differences between AS and NA HBF (*p* < 0.05).

**Table 4:** Reactivity of myofibroblasts derived from NA and AS HBF to an electric field.

3 V/cm dcEF; FMT (TGF-β 5 ng/mL for 5 days); *n* = 20	Speed of Cell Migration [μm/min]	Cell Displacement [μm]	Directionality (cos γ) [No Unit]	Orientation Index [No Unit]
Cell Population	Mean ± SEM	Mean ± SEM	Mean ± SEM	Mean ± SEM
NA HBF	0.196 ± 0.026 *	59.106 ± 10.895	0.136 ± 0.131 *	0.357 ± 0.133 *
AS HBF	0.197 ± 0.022	55.431 ± 9.580	0.050 ± 0.118	0.438 ± 0.124

* statistically significant differences between myofibroblasts and undifferentiated cells (*p* < 0.05).

## Data Availability

All data supporting reported results are available upon request from the corresponding authors.

## References

[1] du Bois-Reymond E. (1848). Untersuchungen Über Thierische Elektricität.

[2] McCaig C.D., Rajnicek A.M., Song B., Zhao M. (2005). Controlling Cell Behavior Electrically: Current Views and Future Potential. Physiol. Rev..

[3] Zhao M. (2009). Electrical Fields in Wound Healing—An Overriding Signal That Directs Cell Migration. Semin. Cell Dev. Biol..

[4] Djamgoz M.B.A., Mycielska M., Madeja Z., Fraser S.P., Korohoda W. (2001). Directional Movement of Rat Prostate Cancer Cells in Direct-Current Electric Field: Involvement of Voltagegated Na+ Channel Activity. J. Cell Sci..

[5] Mycielska M.E., Djamgoz M.B.A. (2004). Cellular Mechanisms of Direct-Current Electric Field Effects: Galvanotaxis and Metastatic Disease. J. Cell Sci..

[6] Sulik G.L., Soong H.K., Chang P.C., Parkinson W.C., Elner S.G., Elner V.M. (1992). Effects of Steady Electric Fields on Human Retinal Pigment Epithelial Cell Orientation and Migration in Culture. Acta Ophthalmol..

[7] Trollinger D.R., Isseroff R.R., Nuccitelli R. (2002). Calcium Channel Blockers Inhibit Galvanotaxis in Human Keratinocytes. J. Cell Physiol..

[8] Fang K.S., Farboud B., Nuccitelli R., Isseroff R.R. (1998). Migration of Human Keratinocytes in Electric Fields Requires Growth Factors and Extracellular Calcium. J. Investig. Dermatol..

[9] Nishimura K.Y., Isseroff R.R., Nucciteili R., Nuccitelli R. (1996). Human Keratinocytes Migrate to the Negative Pole in Direct Current Electric Fields Comparable to Those Measured in Mammalian Wounds. J. Cell Sci..

[10] Sheridan D.M., Isseroff R.R., Nuccitelli R. (1996). Imposition of a Physiologic DC Electric Field Alters the Migratory Response of Human Keratinocytes on Extracellular Matrix Molecules. J. Investig. Dermatol..

[11] Soong H.K., Parkinson W.C., Bafna S., Sulik G.L., Huang S.C. (1990). Movements of Cultured Corneal Epithelial Cells and Stromal Fibroblasts in Electric Fields. Investig. Ophthalmol. Vis. Sci..

[12] Farboud B., Nuccitelli R., Schwab I.R., Isseroff R.R. (2000). DC Electric Fields Induce Rapid Directional Migration in Cultured Human Corneal Epithelial Cells. Exp. Eye Res..

[13] Zhao M., Agius-Fernandez A., Forrester J.V., McCaig C.D. (1996). Directed Migration of Corneal Epithelial Sheets in Physiological Electric Fields. Investig. Ophthalmol. Vis. Sci..

[14] Li X., Kolega J. (2002). Effects of Direct Current Electric Fields on Cell Migration and Actin Filament Distribution in Bovine Vascular Endothelial Cells. J. Vasc. Res..

[15] Kim M.S., Lee M.H., Kwon B.-J., Seo H.J., Koo M.-A., You K.E., Kim D., Park J.-C. (2017). Control of Neonatal Human Dermal Fibroblast Migration on Poly(Lactic-Co-Glycolic Acid)-Coated Surfaces by Electrotaxis. J. Tissue Eng. Regen. Med..

[16] Stump R.F., Robinson K.R. (1983). *Xenopus* Neural Crest Cell Migration in an Applied Electrical Field. J. Cell Biol..

[17] Nuccitelli R., Smart T. (1989). Extracellular Calcium Levels Strongly Influence Neural Crest Cell Galvanotaxis. Biol. Bull..

[18] Cooper M.S., Keller R.E. (1984). Perpendicular Orientation and Directional Migration of Amphibian Neural Crest Cells in Dc Electrical Fields. Proc. Natl. Acad. Sci. USA.

[19] Allen G.M., Mogilner A., Theriot J.A. (2013). Electrophoresis of Cellular Membrane Components Creates the Directional Cue Guiding Keratocyte Galvanotaxis. Curr. Biol..

[20] Cooper M.S., Schliwa M. (1985). Electrical and Ionic Controls of Tissue Cell Locomotion in DC Electric Fields. J. Neurosci. Res..

[21] Cooper M.S., Schliwa M. (1986). Motility of Cultured Fish Epidermal Cells in the Presence and Absence of Direct Current Electric Fields. J. Cell Biol..

[22] Sroka J., Krecioch I., Zimolag E., Lasota S., Rak M., Kedracka-Krok S., Borowicz P., Gajek M., Madeja Z. (2016). Lamellipodia and Membrane Blebs Drive Efficient Electrotactic Migration of Rat Walker Carcinosarcoma Cells WC 256. PLoS ONE.

[23] Ferrier J., Ross S.M., Kanehisa J., Aubin J.E. (1986). Osteoclasts and Osteoblasts Migrate in Opposite Directions in Response to a Constant Electrical Field. J. Cell Physiol..

[24] Orida N., Feldman J.D. (1982). Directional Protrusive Pseudopodial Activity and Motility in Macrophages Induced by Extracellular Electric Fields. Cell Motil..

[25] Rapp B., de Boisfleury-Chevance A., Gruler H. (1988). Galvanotaxis of Human Granulocytes. Dose-Response Curve. Eur. Biophys. J..

[26] Zhang J., Calafiore M., Zeng Q., Zhang X., Huang Y., Li R.A., Deng W., Zhao M. (2011). Electrically Guiding Migration of Human Induced Pluripotent Stem Cells. Stem Cell Rev..

[27] Bai H., McCaig C.D., Forrester J.V., Zhao M. (2004). DC Electric Fields Induce Distinct Preangiogenic Responses in Microvascular and Macrovascular Cells. Arterioscler. Thromb. Vasc. Biol..

[28] Nuccitelli R. (2003). A Role for Endogenous Electric Fields in Wound Healing. Curr. Top Dev. Biol..

[29] McCaig C.D., Song B., Rajnicek A.M. (2009). Electrical Dimensions in Cell Science. J. Cell Sci..

[30] Sorg H., Tilkorn D.J., Hager S., Hauser J., Mirastschijski U. (2017). Skin Wound Healing: An Update on the Current Knowledge and Concepts. Eur. Surg. Res..

[31] Yang J., Liu X., Wang W., Chen Y., Liu J., Zhang Z., Wu C., Jiang X., Liang Y., Zhang J.P. (2022). Bioelectric Fields Coordinate Wound Contraction and Re-Epithelialization Process to Accelerate Wound Healing via Promoting Myofibroblast Transformation. Bioelectrochemistry.

[32] Sun Y.-H.Y.-H., Reid B., Fontaine J.H., Miller L., Hyde D.M., Mogilner A., Zhao M. (2011). Airway Epithelial Wounds in Rhesus Monkey Generate Ionic Currents That Guide Cell Migration to Promote Healing. J. Appl. Physiol. (1985).

[33] Nadel J.A., Davis B., Phipps R.J. (1979). Control of Mucus Secretion and Ion Transport in Airways. Annu. Rev. Physiol..

[34] Tomkiewicz R.P., Albers G.M., De Sanctis T., Ramirez O.E., King M., Rubin B.K. (1995). Species Differences in the Physical and Transport Properties of Airway Secretions. Can. J. Physiol. Pharmacol..

[35] Park J.A., Kim J.H., Bi D., Mitchel J.A., Qazvini N.T., Tantisira K., Park C.Y., McGill M., Kim S.H., Gweon B. (2015). Unjamming and Cell Shape in the Asthmatic Airway Epithelium. Nat. Mater..

[36] Michalik M., Wójcik-Pszczoła K., Paw M., Wnuk D., Koczurkiewicz P., Sanak M., Pękala E., Madeja Z. (2018). Fibroblast-to-Myofibroblast Transition in Bronchial Asthma. Cell. Mol. Life Sci..

[37] Hackett T.L., Knight D.A. (2007). The Role of Epithelial Injury and Repair in the Origins of Asthma. Curr. Opin. Allergy Clin. Immunol..

[38] Inoue H., Akimoto K., Homma T., Tanaka A., Sagara H. (2020). Airway Epithelial Dysfunction in Asthma: Relevant to Epidermal Growth Factor Receptors and Airway Epithelial Cells. J. Clin. Med..

[39] Roth H.M., Wadsworth S.J., Kahn M., Knight D.A. (2012). The Airway Epithelium in Asthma: Developmental Issues That Scar the Airways for Life?. Pulm. Pharmacol. Ther..

[40] Davies D.E., Wicks J., Powell R.M., Puddicombe S.M., Holgate S.T. (2003). Airway Remodeling in Asthma: New Insights. J. Allergy Clin. Immunol..

[41] Wnuk D., Lasota S., Paw M., Madeja Z., Michalik M. (2020). Asthma-Derived Fibroblast to Myofibroblast Transition Is Enhanced in Comparison to Fibroblasts Derived from Non-Asthmatic Patients in 3D in Vitro Culture Due to Smad2/3 Signalling. Acta Biochim. Pol..

[42] Wnuk D., Paw M., Ryczek K., Bochenek G., Sładek K., Madeja Z., Michalik M. (2020). Enhanced Asthma-Related Fibroblast to Myofibroblast Transition Is the Result of Profibrotic TGF-β/Smad2/3 Pathway Intensification and Antifibrotic TGF-β/Smad1/5/(8)9 Pathway Impairment. Sci. Rep..

[43] Paw M., Borek I., Wnuk D., Ryszawy D., Piwowarczyk K., Kmiotek K., Wojcik-Pszczoła K.A., Pierzchalska M., Madeja Z., Sanak M. (2017). Connexin43 Controls the Myofibroblastic Differentiation of Bronchial Fibroblasts from Patients with Asthma. Am. J. Respir. Cell. Mol. Biol..

[44] Michalik M., Pierzchalska M., Włodarczyk A., Wójcik K.A., Czy J., Sanak M., Madeja Z. (2011). Transition of Asthmatic Bronchial Fibroblasts to Myofibroblasts Is Inhibited by Cell-Cell Contacts. Respir. Med..

[45] Michalik M., Pierzchalska M., Legutko A., Ura M., Ostaszewska A., Soja J., Sanak M. (2009). Asthmatic Bronchial Fibroblasts Demonstrate Enhanced Potential to Differentiate into Myofibroblasts in Culture. Med. Sci. Monit..

[46] Paw M., Wnuk D., Jakieła B., Bochenek G., Sładek K., Madeja Z., Michalik M. (2021). Responsiveness of Human Bronchial Fibroblasts and Epithelial Cells from Asthmatic and Non-Asthmatic Donors to the Transforming Growth Factor-Β1 in Epithelial-Mesenchymal Trophic Unit Model. BMC Mol. Cell. Biol..

[47] Sarna M., Wojcik K.A., Hermanowicz P., Wnuk D., Burda K., Sanak M., Czyö J., Michalik M. (2015). Undifferentiated Bronchial Fibroblasts Derived from Asthmatic Patients Display Higher Elastic Modulus than Their Non-Asthmatic Counterparts. PLoS ONE.

[48] Wójcik K.A., Skoda M., Koczurkiewicz P., Sanak M., Czyz J., Michalik M. (2013). Apigenin Inhibits TGF-Β1 Induced Fibroblast-to-Myofibroblast Transition in Human Lung Fibroblast Populations. Pharmacol. Rep..

[49] Sroka J., Zimolag E., Lasota S., Korohoda W., Madeja Z., Gautreau A. (2018). Electrotaxis: Cell Directional Movement in Electric Fields. Cell Migration.

[50] Krecioch I., Madeja Z., Lasota S., Zimolag E., Sroka J. (2015). The Role of Microtubules in Electrotaxis of Rat Walker Carcinosarcoma WC256 Cells. Acta Biochim. Pol..

[51] Zimolag E., Borowczyk-Michalowska J., Kedracka-Krok S., Skupien-Rabian B., Karnas E., Lasota S., Sroka J., Drukala J., Madeja Z. (2017). Electric Field as a Potential Directional Cue in Homing of Bone Marrow-Derived Mesenchymal Stem Cells to Cutaneous Wounds. Biochim. Et Biophys. Acta (BBA) -Mol. Cell Res..

[52] Schindelin J., Arganda-Carreras I., Frise E., Kaynig V., Longair M., Pietzsch T., Preibisch S., Rueden C., Saalfeld S., Schmid B. (2012). Fiji: An Open-Source Platform for Biological-Image Analysis. Nat. Methods.

[53] Paw M., Wnuk D., Nit K., Bobis-Wozowicz S., Szychowski R., Ślusarczyk A., Madeja Z., Michalik M. (2021). SB203580-A Potent P38 MAPK Inhibitor Reduces the Profibrotic Bronchial Fibroblasts Transition Associated with Asthma. Int. J. Mol. Sci..

[54] Chang H.-F., Cheng H.-T., Chen H.-Y., Yeung W.K., Cheng J.-Y. (2019). Doxycycline Inhibits Electric Field-Induced Migration of Non-Small Cell Lung Cancer (NSCLC) Cells. Sci. Rep..

[55] Frederich B.J., Timofeyev V., Thai P.N., Haddad M.J., Poe A.J., Lau V.C., Moshref M., Knowlton A.A., Sirish P., Chiamvimonvat N. (2017). Electrotaxis of Cardiac Progenitor Cells, Cardiac Fibroblasts, and Induced Pluripotent Stem Cell-Derived Cardiac Progenitor Cells Requires Serum and Is Directed via PI3′K Pathways. Heart Rhythm.

[56] Guo A., Song B., Reid B., Gu Y., Forrester J.V., Jahoda C.A.B., Zhao M. (2010). Effects of Physiological Electric Fields on Migration of Human Dermal Fibroblasts. J. Investig. Dermatol..

[57] Liu J., Guo X., Ren X., Tian H., Liang Y., Luo Z., Wang W., Wang Y., Zhang D., Huang Y. (2018). A Novel FPCL Model Producing Directional Contraction through Induction of Fibroblast Alignment by Biphasic Pulse Direct Current Electric Field. Exp. Cell Res..

[58] Onuma E.K., Hui S.W. (1988). Electric Field-Directed Cell Shape Changes, Displacement, and Cytoskeletal Reorganization Are Calcium Dependent. J. Cell Biol..

[59] Finkelstein E., Chang W., Chao P.-H.G., Gruber D., Minden A., Hung C.T., Bulinski J.C. (2004). Roles of Microtubules, Cell Polarity and Adhesion in Electric-Field-Mediated Motility of 3T3 Fibroblasts. J. Cell Sci..

[60] Huang Y.-J., Samorajski J., Kreimer R., Searson P.C. (2013). The Influence of Electric Field and Confinement on Cell Motility. PLoS ONE.

[61] Brown M.J., Loew L.M. (1994). Electric Field-Directed Fibroblast Locomotion Involves Cell Surface Molecular Reorganization and Is Calcium Independent. J. Cell Biol..

[62] Guo L., Xu C., Li D., Zheng X., Tang J., Bu J., Sun H., Yang Z., Sun W., Yu X. (2015). Calcium Ion Flow Permeates Cells through SOCs to Promote Cathode-Directed Galvanotaxis. PLoS ONE.

[63] Erickson C.A., Nuccitelli R. (1984). Embryonic Fibroblast Motility and Orientation Can Be Influenced by Physiological Electric Fields. J. Cell Biol..

[64] Ross S.M., Ferrier J.M., Aubin J.E. (1989). Studies on the Alignment of Fibroblasts in Uniform Applied Electrical Fields. Bioelectromagnetics.

[65] Sillman A.L., Quang D.M., Farboud B., Fang K.S., Nuccitelli R., Isseroff R.R. (2003). Human Dermal Fibroblasts Do Not Exhibit Directional Migration on Collagen I in Direct-Current Electric Fields of Physiological Strength. Exp. Dermatol..

[66] Zhao M., Dick A., Forrester J.V., McCaig C.D. (1999). Electric Field-Directed Cell Motility Involves up-Regulated Expression and Asymmetric Redistribution of the Epidermal Growth Factor Receptors and Is Enhanced by Fibronectin and Laminin. Mol. Biol. Cell.

[67] Li F., Wang H., Li L., Huang C., Lin J., Zhu G., Chen Z., Wu N., Feng H. (2012). Superoxide Plays Critical Roles in Electrotaxis of Fibrosarcoma Cells via Activation of ERK and Reorganization of the Cytoskeleton. Free Radic. Biol. Med..

[68] Li F., Chen T., Hu S., Lin J., Hu R., Feng H. (2013). Superoxide Mediates Direct Current Electric Field-Induced Directional Migration of Glioma Cells through the Activation of AKT and ERK. PLoS ONE.

[69] Sun S., Wise J., Cho M. (2004). Human Fibroblast Migration in Three-Dimensional Collagen Gel in Response to Noninvasive Electrical Stimulus. I. Characterization of Induced Three-Dimensional Cell Movement. Tissue Eng..

